# Fit-Ideal Internalisation and Disordered Eating in Adolescent Girls: Psychometric Evaluation and Incremental Validity of the Fit Internalization Test

**DOI:** 10.3390/nu18193248

**Published:** 2026-10-01

**Authors:** Migle Baceviciene, Vaiva Balciuniene, Rasa Jankauskiene

**Affiliations:** 1 Department of Physical and Social Education, Lithuanian Sports University, Sporto 6, 44221 Kaunas, Lithuania; vaiva.balciuniene@lsu.lt; 2 Institute of Sport Science and Innovations, Lithuanian Sports University, Sporto 6, 44221 Kaunas, Lithuania; rasa.jankauskiene@lsu.lt

**Keywords:** fit ideal internalization, body image, adolescent girls, psychometric validation, measurement invariance, sports, disordered eating

## Abstract

Objectives: The present study aimed to validate the Lithuanian translation of the Fit Ideal Internalization Test (FIIT) in adolescent girls. Methods: A total of 1067 girls (mean age = 17.27, SD = 0.61; age range = 15–19 years) participated in the study. FIIT, muscular ideal internalization, body dissatisfaction (BD), disordered eating (DE), body appreciation (BA), and exercise motivation were assessed. Results: Exploratory factor analysis (*n* = 534) replicated the original three-factor structure, explaining 74.43% of the total variance. Confirmatory factor analysis (*n* = 533) supported the three-factor model, with strong factor loadings. Composite reliability was excellent (McDonald’s ω = 0.919–0.969). Measurement invariance was established across sport participation groups, and sport-involved girls reported higher Fit Drive, Fit Overvaluation, and FIIT total scores than non-sport-involved girls. All FIIT subscales were positively associated with muscular internalization, BD, and DE and negatively associated with BA, while Fit Idealisation and Fit Overvaluation showed weak negative associations with autonomous exercise motivation, consistent with the hypothesised pattern of associations. The FIIT explained significant additional variance in DE beyond body mass index, BD, and muscular ideal internalization, with Fit Idealisation showing the strongest unique association (β = 0.401, *p* < 0.001). Conclusions: The Lithuanian FIIT appears to be a psychometrically sound instrument for assessing fit ideal internalization among Lithuanian adolescent girls.

## 1. Introduction

Negative body image is characterised by persistent dissatisfaction, negative evaluations, and distress related to one’s body, weight, shape, or physical appearance. It encompasses a person’s perceptions, thoughts, and feelings about their body, which may be distorted or disproportionately negative and are often accompanied by body shame, self-objectification, and frequent comparisons with internalised sociocultural appearance ideals [1,2,3]. Body image concerns among adolescent girls and young women represent a significant public health issue. A substantial body of evidence has demonstrated associations between body image concerns and a range of adverse outcomes, including psychological distress, low self-esteem, sport attrition, compulsive exercise, disordered eating, and clinical eating disorders [4,5,6,7,8,9,10].

Sociocultural models have consistently identified the internalisation of idealised body standards as a key mechanism through which sociocultural pressures, including media influences, contribute to body dissatisfaction and disordered eating [11,12,13,14,15,16,17]. For decades, research on body ideal internalisation among women has primarily focused on the thin ideal—the culturally prescribed standard that equates female attractiveness with extreme thinness [2,18]. However, the landscape of idealised female body standards has evolved considerably over time, with increasing emphasis on a physique that is not only thin but also toned, strong, and athletic [19,20]. The widespread use of social media platforms has further amplified exposure to these appearance ideals, particularly among young people, by providing continuous access to idealized body-related content, unlimited opportunities for photo and selfie manipulation and appearance-based comparisons [17,21,22].

Contemporary media environments, particularly highly visual social media platforms such as Instagram, TikTok, and Snapchat, extensively promote the fit or athletic ideal as the contemporary standard of female beauty and health. This ideal emphasises a physique that is simultaneously lean, toned, strong, and physically fit [19,23]. Content analyses of fitspiration (“fit inspiration”) imagery have shown that such posts frequently combine objectifying depictions of lean, muscular, and sexualised women with motivational messages related to diet and exercise. These messages often contain elements of guilt and self-discipline, reinforcing both the desirability and perceived attainability of a body characterised by low body fat and visible muscle definition [19,23,24,25,26,27]. Paradoxically, although fitspiration content is intended to promote exercise and healthy lifestyle behaviours, the images typically depict static, posed bodies rather than the physical activities themselves. Moreover, evidence from a large-scale study indicated that women are more frequent consumers of fitspiration content than men [28].

Some data was provided reporting that fitspiration might be a healthier alternative to thinspiration [26]. However, an increasing body of research suggests that the effects of fitspiration may not be as beneficial as initially assumed. A systematic review concluded that exposure to fitspiration content is associated with negative body image outcomes, particularly among younger populations who are more frequently exposed to such content [29]. Exposure to fit ideal imagery has been shown to produce greater body dissatisfaction than exposure to the traditional thin ideal, suggesting that the fit ideal may represent an even more discrepant and therefore more psychologically harmful standard [30,31,32]. Further research also showed that internalization of fit ideals leads women to compare themselves to others, feel dissatisfied with their bodies and to engage in unhealthy eating and exercising behaviours in the same way as thin ideal internalization does [33,34]. Analyses of fitspiration-related social media content have further demonstrated that messages promoting appearance-based body ideals and weight-management behaviours are more prevalent than those emphasising health and well-being, with attractiveness frequently presented as the primary motivation for engaging in such behaviours [35]. Consequently, fit ideal internalization may encourage participation in physical activity that is driven by appearance concerns and external pressures rather than by autonomous exercise motivation.

Despite the growing literature documenting the prevalence and consequences of fit ideal exposure, research on the internalization of this body standard has been substantially constrained by the absence of a validated measurement instrument. Existing measures of body ideal internalization—most notably the Sociocultural Attitudes Toward Appearance Questionnaire SATAQ-4R [15]—assess thin and muscular internalization as separate constructs. However, neither dimension adequately captures fit ideal internalization, which is conceptually defined by the simultaneous pursuit of thinness and toned muscularity rather than either appearance standard in isolation. This limitation has restricted researchers’ ability to examine fit ideal internalization as a distinct construct and to investigate its unique associations with body image, eating behaviours, and related psychological outcomes.

To address this gap, Uhlmann et al. [36] developed and validated the Fit Ideal Internalization Test (FIIT)—a 20-item self-report instrument designed to comprehensively assess women’s internalization of the fit ideal across three dimensions [36]. Drawing on internalization theory [12,16], the FIIT operationalizes fit ideal internalization as comprising three related dimensions: (1) fit idealisation, reflecting the acceptance of the socially endorsed fit body standard as one’s own and the desire to embody this standard (e.g., “I am preoccupied with the idea of having a body that looks both lean and toned”); (2) fit overvaluation, reflecting the extent to which achieving a lean and toned body is perceived as personally and socially meaningful, such that the fit ideal is linked to self-worth, discipline, and life success (e.g., “Having a body that is both lean and toned is a good way to gain respect from other people”); and (3) fit behavioural drive, reflecting engagement in behaviours aimed at achieving the fit ideal, such as exercise, dieting, and supplement use (e.g., “I spend time doing things to ensure my body looks both lean and toned”). Fit idealization and fit overvaluation subscales consist of 8 items, and fit drive subscale includes 4 items. Respondents rate each item on a 5-point Likert-type scale ranging from 1 (strongly disagree) to 5 (strongly agree), with higher scores indicating greater fit ideal internalization. Subscale scores are calculated as the mean of the corresponding items, and the total FIIT score is calculated as the mean across all 20 items. Across three studies conducted with Australian university women, the original FIIT demonstrated excellent psychometric properties, including good internal consistency (Cronbach’s α = 0.90–0.93 for subscales and α = 0.94–0.95 for the total score), confirmatory factor-analytic support for the three-factor structure, strong convergent and discriminant validity, incremental validity above and beyond existing measures of thin and muscular internalization, and high two-week test–retest reliability [36].

Given the cross-cultural spread of social media and the global reach of Western appearance ideals, there is a clear need to validate the FIIT across diverse linguistic and cultural contexts, particularly among younger populations who are especially susceptible to social media influences. Adolescent girls represent a particularly vulnerable group, as body dissatisfaction, disordered eating, and related body image concerns tend to peak during adolescence and early adulthood [37]. Moreover, social media use among adolescents is nearly universal in most Western countries [38], including Lithuania [39]. Lithuania represents a relevant context for body image research. As an Eastern European country undergoing rapid socioeconomic and cultural transformation, alongside the growth of the commercial fitness sector and increasing exposure to health-, fitness-, and appearance-oriented lifestyles, Lithuania is experiencing significant concerns related to body image and disordered eating among young people [40,41]. However, to date, no validated Lithuanian instrument exists for assessing fit ideal internalization, limiting research, cross-cultural comparisons, and evidence-informed prevention efforts in this area.

The close association of the fit ideal with physical activity and fitness also raises the question of whether FIIT items function similarly among girls with different levels of sport involvement. Sport participation may shape how women and girls interpret fit ideal-related messages and body-related values. Compared with their non-sport-involved peers, sport-involved girls are more likely to value their bodies in terms of body functionality [42]. Consequently, concepts such as being “fit,” “strong,” or “toned” may not exclusively reflect appearance-based body ideals but may also be associated with functional and performance-related attributes. In contrast, non-sport-involved girls may be more likely to interpret these ideals through an appearance-oriented lens. These potential differences raise an important question regarding whether FIIT items function equivalently across sport participation groups. Establishing measurement invariance is therefore necessary to ensure that the construct is interpreted similarly and that comparisons between sport-involved and non-sport-involved adolescent girls are meaningful and unbiased.

The aim of the present study is to develop and validate the Lithuanian translation of the FIIT in a large sample of adolescent girls. Specifically, the study sought to: (1) evaluate the factorial structure of the Lithuanian FIIT through exploratory and confirmatory factor analyses conducted in split-half subsamples; (2) assess composite reliability; (3) examine measurement invariance of the FIIT across sport participation groups; (4) examine whether FIIT scores show theoretically expected associations with muscular ideal internalisation, body dissatisfaction, disordered eating, body appreciation, autonomous exercise motivation, and body mass index (BMI); and (5) test the incremental validity of the FIIT subscales in explaining additional variance in disordered eating symptoms beyond established correlates.

Based on the original validation study [36] and the broader body image literature, we hypothesized that: (1) the three-factor structure of the original FIIT would be replicated in the Lithuanian sample; (2) the FIIT subscales would demonstrate excellent composite reliability; (3) FIIT subscale scores would be positively correlated with muscular internalization, body dissatisfaction, and disordered eating, and negatively correlated with body appreciation and autonomous exercise motivation; and (4) the FIIT subscales would explain unique variance in disordered eating beyond BMI, body dissatisfaction, and muscular internalization. No specific hypothesis was formulated regarding measurement invariance across sport participation groups.

## 2. Materials and Methods

### 2.1. Participants

Participants were recruited from 68 general Lithuanian-speaking schools selected based on established collaboration with the research team and willingness to participate. Eligible participants were 11th-grade students attending these schools; no additional inclusion or exclusion criteria were applied. Of 2015 eligible students invited to participate, 92 declined participation and 1923 completed the survey, corresponding to an overall response rate of 95.4%. Sex was not recorded for students who declined participation; therefore, a sex-specific response rate could not be calculated. The present validation study included the 1067 girls who completed the survey. All questionnaire items were mandatory; therefore, there were no missing questionnaire data. Four cases with implausibly high BMI values indicative of potential data-entry errors were excluded from all analyses involving BMI.

Of the total sample, 266 girls (24.9%) reported no participation in organized sports, whereas 801 girls (75.1%) reported regular participation in organized sport. Body mass index (BMI) ranged from 15.43 to 40.49 kg/m^2^ (M = 21.10, SD = 3.25). Most participants were classified as having a healthy body weight (74.0%), whereas 8.7% were overweight, 2.4% were obese, and 14.9% were underweight.

### 2.2. Procedures

The present study was conducted as part of a larger research project examining body image, physical activity, and health-related behaviours among Lithuanian adolescents. Data were collected between February and May 2022 from secondary schools across Lithuania. Following approval from school administrations, parents or legal guardians were contacted and asked to provide informed consent for their children’s participation in the study. Only students with parental or guardian consent were invited to participate. Data were collected using an online questionnaire administered through SurveyMonkey (www.surveymonkey.com, accessed on 24 August 2026). Trained researcher assistants working under the supervision of the research team provided the survey link to eligible students in their classrooms before or after lessons. Participants completed the questionnaire online at their own pace, with no time limit imposed; completion typically required 35–45 min. Prior to accessing the questionnaire, participants were presented with information about the study and asked to provide digital informed consent. Those who consented were directed to the survey measures described below. Participation was voluntary, and participants could withdraw at any time by closing their browser window without submitting any responses.

### 2.3. Test Adaptation

The translation and cultural adaptation of the FIIT followed established cross-cultural validation procedures [43]. First, the original FIIT was independently translated into Lithuanian by two bilingual translators. The two translations were then compared and synthesized into a single preliminary version, with any discrepancies resolved through discussion and consideration of the translators’ reports. Next, the preliminary Lithuanian version was independently back-translated into English by two bilingual translators who were unfamiliar with the original instrument. The original, translated, and back-translated versions were then reviewed by experts in body image research to evaluate semantic and conceptual equivalence, cultural appropriateness, and linguistic clarity. The expert review confirmed that the Lithuanian translation adequately reflected the meaning of the original items, and no further modifications were deemed necessary.

To assess item comprehensibility and relevance for the target population, the preliminary Lithuanian version was pilot-tested with several adolescent girls from the intended age group. Participants were asked to complete the questionnaire and provide feedback regarding item clarity, wording, and interpretation. Based on their feedback, one linguistic modification was made. Specifically, the expression „stangrus kūnas” (corresponding to “toned body”), initially proposed during the translation process, was perceived as unclear and unnatural by the adolescents and was therefore replaced with „tvirtas kūnas”, which was considered more familiar and understandable to contemporary Lithuanian adolescents. No other substantive modifications were required. The final Lithuanian version of the FIIT is provided in Appendix A.

### 2.4. Measures

Participants provided demographic information, including age, gender, school and sport participation.

Sport participation was assessed using self-reported questions. Participants indicated whether they regularly participated in competitive/achievement sport or in leisure-time exercise. Competitive/achievement sport was defined as regular active participation in sport involving competition, whereas leisure-time exercise was defined as participation in sport or physical activity without the intention to compete. Participants reporting either competitive/achievement sport or leisure-time exercise were classified as participating in sport (“yes”), whereas those reporting neither were classified as non-participants (“no”).

The Lithuanian translated version (FIIT) of the FIIT original [36] was used in the present study. The original FIIT consists of 20 items rated on a 5-point Likert scale ranging from 1 (strongly disagree) to 5 (strongly agree).

Muscular Ideal Internalization was assessed using the muscular internalization subscale of the Sociocultural Attitudes Toward Appearance Questionnaire–4 (SATAQ-4) [14]. The Internalization: Muscular/Athletic subscale of the SATAQ-4 assesses the extent to which individuals internalize athletic and muscular appearance ideals (e.g., “I think a lot about looking muscular” and “I spend a lot of time doing things to look more athletic”). Participants rated their agreement with each item on a 5-point scale (1 = definitely disagree to 5 = definitely agree). The psychometric properties of the Lithuanian translation of the instrument were good [40]. In the present sample, the composite reliability (McDonald’s ω) was excellent: 0.936 (95% CI: 0.927, 0.943).

Body dissatisfaction was assessed using the Body Dissatisfaction subscale of the Eating Disorder Inventory–3 (EDI-3; [44]. The 10-item subscale measures dissatisfaction with overall body shape and with specific body regions that are commonly associated with body image concerns, such as the stomach, hips, thighs, and buttocks. Participants rated how often each statement applied to them using a 6-point Likert-type scale. Higher scores indicate greater body dissatisfaction. The Lithuanian translation of the scale demonstrated good psychometric properties [45]. In the present sample, the composite reliability (McDonald’s ω) was good: 0.878 (95% CI: 0.865, 0.881).

Disordered eating symptoms were assessed using the Eating Disorder Examination Questionnaire (EDE-Q 6.0; [46]. The questionnaire assesses the severity of eating disorder psychopathology during the preceding 28 days, including concerns about eating, body shape, and weight, as well as restrictive eating attitudes. Example items include “Has your shape influenced how you think about yourself as a person?” and “Have you had a definite desire to have an empty stomach with the aim of influencing your shape or weight?” Participants rated the frequency or severity of eating disorder-related cognitions and behaviours on a 7-point scale ranging from 0 to 6, with higher scores indicating greater symptom severity. The global EDE-Q 6.0 score was used as a continuous measure of overall eating disorder psychopathology. Lithuanian translation showed good psychometric properties [47,48]. In the present sample, the composite reliability (McDonald’s ω) was good: 0.887 (95% CI: 0.870, 0.900).

Positive body image was assessed using the Body Appreciation Scale–2 (BAS-2; [49]. The BAS-2 is a 10-item measure of body appreciation, reflecting acceptance of, respect for, and favourable attitudes toward one’s body, as well as protection of one’s body image from unrealistic sociocultural appearance ideals. Example items include “I respect my body” and “I take a positive attitude toward my body”. Participants respond using a 5-point Likert-type scale ranging from 1 (never) to 5 (always). Higher scores indicate greater body appreciation. The Lithuanian translation of the instrument showed good psychometric properties [50]. In the present sample, the composite reliability (McDonald’s ω) was excellent: 0.963 (95% CI: 0.959, 0.967).

Exercise motivation was assessed using the Behavioural Regulation in Exercise Questionnaire–2 (BREQ-2) [51]. Participants rated the extent to which different reasons for exercising (e.g., “I exercise because other people say I should” and “I exercise because it’s fun”) reflected their own motivation on a 5-point Likert-type scale ranging from 0 (not true for me) to 4 (very true for me). A Relative Autonomy Index (RAI) was calculated by weighting each motivational regulation according to its position on the self-determination continuum and summing the weighted scores [(−3 × amotivation) + (−2 × external regulation) + (−1 × introjected regulation) + (2 × identified regulation) + (3 × intrinsic motivation)]. Higher RAI scores indicate a more autonomous and self-determined motivational orientation toward physical activity. The Lithuanian version of the instrument showed good psychometric properties [52].

BMI was calculated from self-reported height and weight (kg/m^2^). Participants were classified as underweight, healthy weight, overweight, or obese according to the age- and sex-specific BMI cut-off points proposed by the [53]. Four cases with implausibly high BMI values indicative of potential data-entry errors were excluded from all analyses involving BMI.

### 2.5. Analytic Strategy

Data treatment. Data analyses were conducted using IBM SPSS Statistics version 31 (IBM Corp., Armonk, NY, USA) and R version 4.6.0 (R Foundation for Statistical Computing, Vienna, Austria). The dataset containing the study variables used in the present analyses is provided in Supplementary Table S1. Descriptive statistics were calculated for all FIIT subscales and the total score, including means, standard deviations, medians, skewness, kurtosis, and score ranges. Next, skewness and kurtosis were examined for all continuous variables included in the correlation and regression analyses. Composite reliability was assessed using McDonald’s omega coefficients. McDonald’s omega was calculated because it is considered a more robust reliability estimate for multidimensional latent constructs than Cronbach’s alpha [54].

The EFA and CFA subsamples. To examine the factorial structure of the Lithuanian version of the FIIT, the total sample was randomly divided into two split-half subsamples using SPSS’s random sample function. Exploratory factor analysis (EFA) was performed in the first subsample (*n* = 534), whereas confirmatory factor analysis (CFA) and subsequent validation procedures were conducted in the second subsample (*n* = 533). Both subsamples exceeded commonly recommended sample-size guidelines for factor analysis, which suggest a minimum of 5–10 participants per item, with larger samples providing more stable factor solutions. Prior to factor analyses, the possible differences between the EFA and CFA subsamples were examined. No significant differences were observed between the subsamples with respect to age, t(1065) = −0.04, *p* = 0.971, BMI, t(1061) = 1.42, *p* = 0.155, or sport participation, χ^2^(1) = 0.69, *p* = 0.406.

Exploratory factor analysis (EFA). The suitability of the data for factor analysis was evaluated using the Kaiser–Meyer–Olkin (KMO) measure of sampling adequacy and Bartlett’s test of sphericity. EFA was conducted using principal axis factoring with Promax rotation and Kaiser normalisation, as correlations between latent factors were expected. The number of retained factors was determined according to the Kaiser criterion (eigenvalues > 1), scree plot inspection, theoretical interpretability, and parallel analysis using polychoric correlations and the 95th-percentile criterion based on 100 simulated datasets.

Confirmatory factor analysis (CFA). Prior to CFA, multivariate normality was evaluated using Mardia’s test with the MVN package in R [55,56]. Because the data demonstrated deviations from multivariate normality, robust estimation methods were applied in subsequent analyses. Mardia’s test indicated significant multivariate skewness (8744.34, *p* < 0.001) and kurtosis (146.23, *p* < 0.001), suggesting violation of the multivariate normality assumption.

CFA and measurement invariance analyses were conducted in R using the lavaan package. Robust maximum likelihood estimation (MLR) was applied because of deviations from multivariate normality. FIIT items were measured using five response categories and were treated as approximately continuous indicators. Given the relatively large CFA sample and evidence supporting the use of robust MLR for ordinal indicators with five or more response categories, MLR was considered appropriate; moreover, MLR provides robust standard errors and scaled test statistics under non-normality [57,58]. Model fit was evaluated using the Satorra–Bentler scaled chi-square statistic (SBχ^2^), robust comparative fit index (CFI), robust Tucker–Lewis index (TLI), robust root mean square error of approximation (RMSEA), and standardised root mean square residual (SRMR). Following recommendations by Hu and Bentler [59], CFI and TLI values ≥ 0.90 indicated acceptable fit and values ≥ 0.95 indicated good fit, whereas RMSEA and SRMR values ≤ 0.08 reflected acceptable model fit. Because the chi-square statistic is sensitive to sample size, greater emphasis was placed on approximate fit indices.

Modification indices were inspected to identify theoretically meaningful localised areas of strain. Residual covariances between items belonging to the same latent factor were added only when conceptually justified and when they substantially improved model fit without altering the theoretical three-factor structure.

To evaluate whether use of the FIIT total score was psychometrically supported, a second-order CFA model was additionally estimated in which Fit Idealisation, Fit Overvaluation, and Fit Drive loaded onto a higher-order general FIIT factor. The second-order model retained the same residual covariances as the final three-factor CFA model and was estimated using MLR.

As a sensitivity analysis, the final three-factor CFA model was additionally estimated treating the five-category FIIT items as ordinal indicators using weighted least squares mean and variance adjusted (WLSMV) estimation based on polychoric correlations.

Measurement Invariance across sport participation groups. Measurement invariance of the final CFA model was examined sequentially by testing configural, metric, scalar, and strict invariance models. Invariance was evaluated using changes in fit indices between nested models. Following the recommended criteria [60], invariance was supported when changes in CFI were ≤0.010, changes in RMSEA were ≤0.015, and changes in SRMR were ≤0.030 for metric invariance and ≤0.015 for scalar and strict invariance.

Differences between girls engaged and not engaged in sports were examined using independent-samples t-tests. Homogeneity of variance was assessed using Levene’s test, and the appropriate t-test estimates were used depending on whether equal variances could be assumed. Effect sizes were estimated using Cohen’s d, with values of 0.20, 0.50, and 0.80 interpreted as small, medium, and large effects, respectively [61].

Further analyses. Theoretically expected associations between FIIT subscale scores and the other study variables were examined using Pearson correlation coefficients. Pearson correlation coefficients were interpreted according to Cohen’s guidelines, where values of 0.10, 0.30, and 0.50 indicated small, moderate, and large associations, respectively [61].

Given that participants were nested within schools, potential school-level clustering was assessed using random-intercept models. Intraclass correlation coefficients (ICCs) were calculated for the FIIT total score and disordered eating, the primary outcome of the incremental validity analysis.

Finally, incremental validity was examined using hierarchical linear regression analyses. Regression assumptions were assessed by examining residual and predicted-value plots, the normal P–P plot, the Durbin–Watson statistic, collinearity diagnostics, and Cook’s distance for influential observations. Because some heteroscedasticity was observed, the robustness of the regression estimates was additionally evaluated using 2000 bootstrap samples with 95% bias-corrected and accelerated (BCa) confidence intervals. Multicollinearity was assessed using the variance inflation factor (VIF) values. In the first step, BMI, muscular internalisation, and body dissatisfaction were entered as predictors of disordered eating. In the second step, the three FIIT subscales were added to determine whether they explained additional variance beyond established body image-related predictors. 

### 2.6. Ethics Approval and Consent to Participate

The study was conducted following the ethical and legal principles of the Declaration of Helsinki, and ethical approval was obtained from the Social Research Ethics Board of Lithuanian Sports University (Approval No. 26005, 5 February 2022). Informed consent was obtained from all participants of the study.

## 3. Results

The descriptive statistics of the FIIT among adolescent girls is presented in Table 1. Mean scores were highest for the Fit Idealisation subscale, followed by the Fit Drive and Fit Overvaluation subscales. Skewness and Kurtosis values for all subscales and the total score were within acceptable ranges, indicating approximately normal distributions.

Exploratory factor analysis (EFA) was conducted in the first split-half subsample (*n* = 534) using Principal Axis Factoring with Promax rotation and Kaiser normalisation. The Kaiser–Meyer–Olkin measure of sampling adequacy was excellent (KMO = 0.969), and Bartlett’s test of sphericity was significant, χ^2^(190) = 11153.45, *p* < 0.001, indicating the suitability of the data for factor analysis. Following the Kaiser criterion (eigenvalues > 1), a three-factor solution was retained, corresponding to Fit Idealisation, Fit Overvaluation, and Fit Drive. Parallel analysis also supported the retention of three factors, providing additional support for the three-factor solution. The retained factors explained 74.43% of the total variance, with Fit Overvaluation accounting for 62.92%, Fit Idealisation for 6.24%, and Fit Drive for 5.26% of the variance. Factor loadings ranged from 0.411 to 0.953, and communalities exceeded 0.55, supporting the adequacy of the retained factor structure (Table 2).

Next, Table 3 presents CFA in the second split-half sample (*n* = 533) and measurement invariance testing across sport participation groups. The initial three-factor CFA model showed acceptable but suboptimal fit. Examination of modification indices suggested several theoretically meaningful residual covariances between items within the same FIIT subscales. Therefore, residual covariances were added between items 3–4, 5–6, 12–13, and 17–18. These residual covariances were theoretically interpretable based on overlapping item content: items 3–4 both reflect cognitive preoccupation with the lean-and-toned body ideal; items 5–6 concern thoughts and behaviours directed toward achieving this ideal; items 12–13 reflect the perceived social and self-presentational meaning of possessing a lean-and-toned physique; and items 17–18 share closely related behavioural content concerning exercise, dieting, and supplement use to achieve specific appearance outcomes. The final CFA model demonstrated improved fit compared with the initial model, with higher robust CFI and TLI values and lower robust RMSEA and SRMR values.

The WLSMV sensitivity analysis treating the FIIT items as ordinal indicators provided additional support for the three-factor structure, with CFI = 0.964, TLI = 0.959, RMSEA = 0.072 (90% CI 0.066–0.078), and SRMR = 0.041. Standardised factor loadings were strong, ranging from 0.787 to 0.923.

A second-order CFA was additionally conducted to examine whether the three FIIT dimensions could be represented by a higher-order general FIIT factor. The model demonstrated acceptable-to-good fit (robust CFI = 0.952, robust TLI = 0.945, robust RMSEA = 0.077, 90% CI 0.068–0.086; SRMR = 0.047). All three first-order factors loaded strongly on the higher-order factor, with standardised loadings of 0.811 for Fit Idealisation, 0.908 for Fit Overvaluation, and 0.871 for Fit Drive. These findings support the presence of a higher-order general FIIT construct underlying the three related dimensions and provide support for use of the total FIIT score as a composite indicator.

The final model was subsequently tested for measurement invariance across sport participation groups. Configural, metric, scalar, and strict invariance were supported, as changes in CFI, RMSEA, and SRMR remained within recommended cut-off values. Although the ΔCFI value for strict invariance reached the recommended threshold (ΔCFI = 0.010), changes in RMSEA and SRMR remained negligible, supporting an overall acceptable level of strict invariance across groups. These findings indicate that the FIIT factor structure and latent construct measurement were largely comparable between girls who were engaged in sports and those who were not.

**Figure 1:**
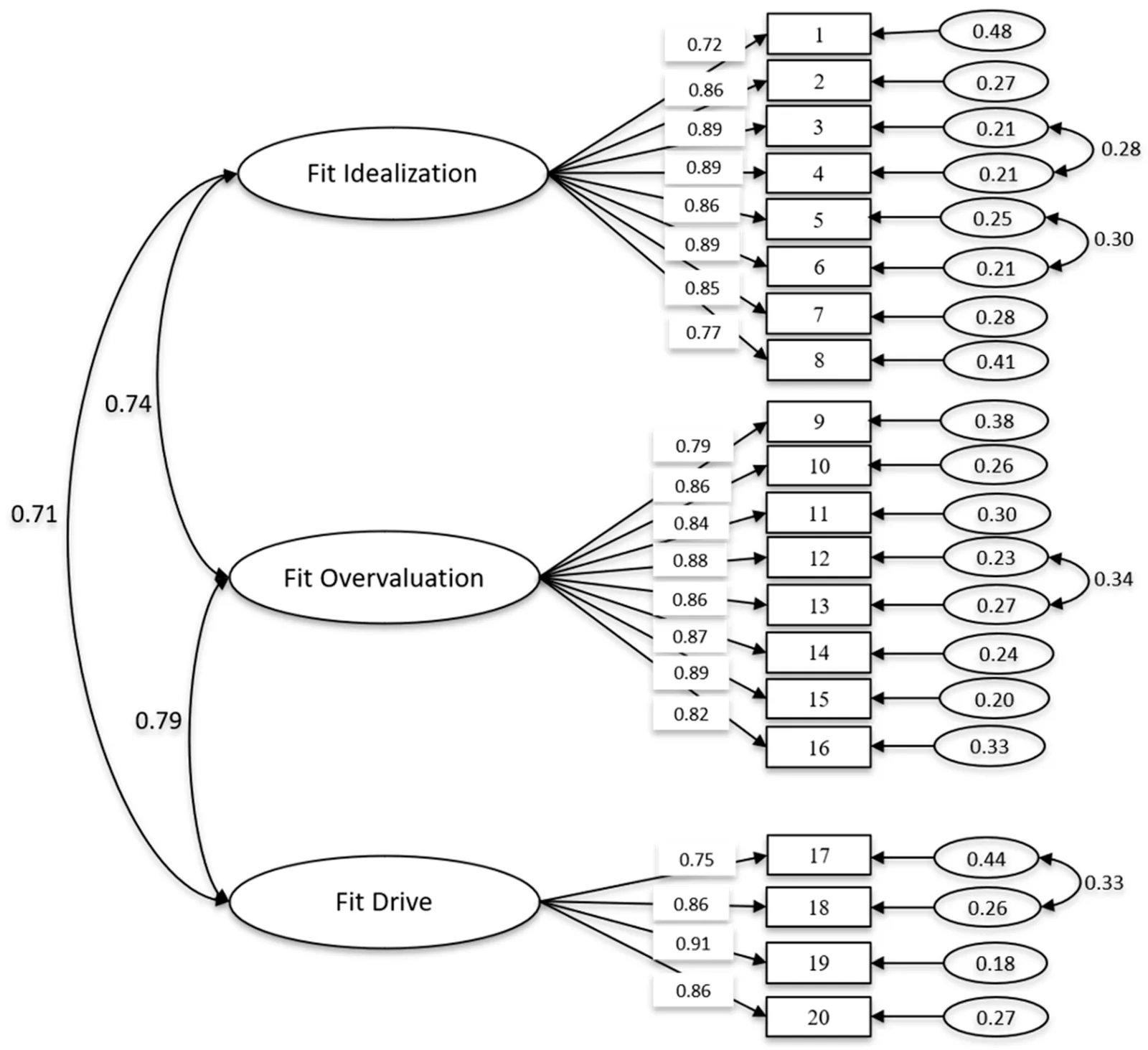
Final three-factor confirmatory factor analysis model of the Fit Ideal Internalisation Test (FIIT) in the second split-half subsample (*n* = 533).

Figure 1 presents the final three-factor confirmatory factor analysis (CFA) model of the FIIT in the second split-half subsample. Standardised factor loadings ranged from 0.72 to 0.91 for Fit Idealisation, from 0.79 to 0.89 for Fit Overvaluation, and from 0.75 to 0.91 for Fit Drive, indicating strong associations between the latent factors and their respective observed indicators. Correlations among the latent factors were moderate to strong, supporting the related but distinct nature of the three FIIT dimensions. Residual covariances were specified between items 3–4, 5–6, 12–13, and 17–18 based on modification indices and conceptual similarity of the items. Overall, the model demonstrated good fit to the data and supported the proposed three-factor structure of the FIIT.

Composite reliability estimates for the FIIT subscales were excellent across the study sample. McDonald’s ω coefficients were 0.952 (95% CI: 0.947, 0.957) for Fit Idealisation, 0.957 (95% CI: 0.951, 0.962) for Fit Overvaluation, and 0.919 (95% CI: 0.907, 0.930) for Fit Drive. The total FIIT score also demonstrated excellent composite reliability, ω = 0.969 (95% CI: 0.966, 0.972). Overall, these findings support the strong reliability of the Lithuanian version of the FIIT.

Girls engaged in sports demonstrated significantly higher scores on Fit Overvaluation, Fit Drive, and the total FIIT score compared with girls not engaged in sports (Table 4). The largest group difference was observed for Fit Drive, with a medium effect size, whereas differences in Fit Overvaluation and the total FIIT score were small. No significant difference was found for Fit Idealisation.

Table 5 presents correlations between the FIIT subscales and study variables. All FIIT dimensions were positively associated with muscular internalisation, body dissatisfaction, and disordered eating symptoms. The strongest correlations were observed between Fit Idealisation and disordered eating, as well as body dissatisfaction. Negative correlations were observed between all FIIT subscales and body appreciation. Relative autonomy demonstrated weak negative associations with Fit Idealisation and Fit Overvaluation, whereas no significant association was found with Fit Drive. Additionally, BMI showed small positive correlations with all FIIT dimensions. Correlations among the FIIT subscales ranged from 0.67 to 0.75, indicating substantial but nonredundant associations between the three constructs.

School-level clustering was low, with ICCs of 0.023 for the FIIT total score and 0.018 for disordered eating, indicating that 2.3% and 1.8% of the variance, respectively, was attributable to between-school differences. Given the minimal school-level clustering, the original individual-level analyses were retained.

Hierarchical regression analysis was conducted to examine the incremental validity of the FIIT subscales in relation to disordered eating symptoms (Table 6). In Step 1, BMI, body dissatisfaction, and muscular internalisation explained 39.3% of the variance in disordered eating. Regression diagnostics indicated acceptable linearity and normality of residuals, independence of errors (Durbin–Watson = 1.948), no problematic multicollinearity (VIF = 1.15–2.99), and no highly influential observations (maximum Cook’s distance = 0.028). Some heteroscedasticity was observed; however, bootstrap analysis based on 2000 samples with 95% BCa confidence intervals yielded the same conclusions regarding statistical significance as the original analysis. Body dissatisfaction showed the strongest independent association with disordered eating, followed by muscular internalisation and BMI. In Step 2, the FIIT subscales were added to the model, resulting in a significant increase in explained variance (ΔR^2^ = 0.092, *p* < 0.001). The final model explained 48.6% of the variance in disordered eating symptoms. Among the FIIT dimensions, only Fit Idealisation showed a significant unique association with disordered eating after controlling for BMI, body dissatisfaction, and muscular internalisation. Fit Overvaluation and Fit Drive did not demonstrate significant unique contributions in the final model. These findings support the incremental validity of the FIIT, particularly the Fit Idealisation dimension, in relation to disordered eating pathology.

## 4. Discussion

The present study aimed to develop and validate the Lithuanian translation of the FIIT [36] in a sample of adolescent girls, extending the cross-cultural reach of this instrument into an Eastern European population. The findings supported the three-factor structure of the original FIIT, demonstrated excellent composite reliability, established measurement invariance across sport participation groups, showed theoretically expected associations with related variables, and provided evidence of incremental validity. To the best of our knowledge, this study represents one of the first cross-cultural validations of the FIIT outside the Australian context. Together, these findings indicate that the Lithuanian FIIT is a psychometrically sound instrument for assessing fit ideal internalization among adolescent girls and contribute to the growing international literature on fit ideal internalization across diverse cultural contexts.

Consistent with the original validation study [36], exploratory factor analysis supported a three-factor structure corresponding to Fit Idealisation, Fit Overvaluation, and Fit Drive. The retained three-factor solution accounted for 74.43% of the total variance, and factor loadings ranged from 0.41 to 0.95, indicating excellent item-level performance. The retained factor solution explained a substantial proportion of variance and demonstrated strong item-factor relationships, providing support for the structural validity of the Lithuanian FIIT. Notably, the factor structure mirrored the theoretically derived conceptualization of fit ideal internalization proposed by Uhlmann et al. [36], suggesting that the cognitive-affective acceptance of the fit ideal, the overvaluation of its personal and social significance, and engagement in fit ideal-congruent behaviours represent distinguishable yet related dimensions of the construct.

The three-factor structure was subsequently confirmed via CFA in the second split-half subsample. The final model demonstrated good fit to the data, supporting the adequacy of the hypothesized measurement model. These fit indices compare favourably with those reported in the original validation study [36] and meet established recommendations for acceptable-to-good model fit [59]. Furthermore, standardized factor loadings were uniformly strong, ranging from 0.72 to 0.91, indicating that the individual items were robust indicators of their respective latent factors. Together, these findings provide further evidence for the structural validity of the Lithuanian FIIT and suggest that the three dimensions of fit ideal internalization identified in the original study are similarly represented among Lithuanian adolescent girls. The replication of the original factor structure across different cultural contexts and age groups strengthens confidence in the theoretical conceptualization of fit ideal internalization as a multidimensional construct.

The successful replication of the three-factor structure in a Lithuanian sample of adolescent girls is theoretically meaningful. However, because the final CFA model included residual covariances identified through modification indices, this partly data-driven model refinement should be independently replicated in future samples.

The present findings support the three-dimensional conceptualisation of fit ideal internalisation proposed by Uhlmann et al. [36] and suggest that it is applicable to Lithuanian adolescent girls. Although further cross-cultural research is needed, the findings provide preliminary evidence that the multidimensional structure of fit ideal internalization extends across different age groups and cultural contexts. Furthermore, the moderate-to-strong inter-factor correlations observed in the present study (r = 0.67–0.75) support the notion that fit idealisation, fit overvaluation, and fit behavioural drive are related yet distinguishable dimensions of fit ideal internalization. Although the correlations were somewhat stronger than those reported in the original validation study (r = 0.48–0.74 in Study 2), the overall pattern was consistent with Uhlmann et al.’s [36] conceptualisation of the three dimensions as interconnected but nonredundant components of a broader fit ideal internalization construct. These findings further support the use of the FIIT as both a multidimensional measure and a composite indicator of fit ideal internalization.

Composite reliability estimates for all FIIT subscales and the total score were high, with McDonald’s omega ranging from 0.919 (Fit Drive) to 0.969 (total score), indicating excellent internal consistency. Given that omega is considered a more robust reliability estimate for multidimensional latent constructs than Cronbach’s alpha [54]. These findings provide strong evidence for the reliability of the Lithuanian FIIT and are consistent with the strong reliability evidence reported in the original validation study, where Cronbach’s alpha coefficients ranged from 0.90 to 0.94 across the three studies [36].

A methodological contribution of the present study was the examination of the measurement invariance of the FIIT across adolescent girls engaged and not engaged in organised sport. Configural, metric, scalar, and strict invariance were all supported, as changes in CFI (≤0.010), RMSEA (≤0.004), and SRMR (≤0.003) remained within the recommended cut-off criteria at each step [60]. These results indicate that the FIIT factor structure, factor loadings, item intercepts, and residual variances are equivalent across sport-involved and non-sport-involved girls, meaning that observed and latent mean score comparisons between these groups are psychometrically justified.

The establishment of measurement invariance across sport participation groups has important implications. Uhlmann et al. [36] noted that their samples were primarily non-athlete university women, leaving open the question of whether the FIIT functions equivalently among physically active women and girls. Sport participation may shape how individuals understand concepts such as being “fit,” “strong,” or “athletic,” as these characteristics may be associated not only with appearance but also with physical competence, health, and sport performance. Consequently, it was plausible that sport-involved and non-sport-involved girls could interpret FIIT items differently. However, support for configural, metric, scalar, and strict invariance indicates that the FIIT assesses fit ideal internalization in a comparable manner across these groups. This finding suggests that differences in FIIT scores between sport-involved and non-sport-involved girls are likely to reflect genuine differences in the underlying construct rather than measurement artefacts. More broadly, it supports the applicability of the FIIT across adolescent populations with varying levels of sport involvement and strengthens confidence in its use for both research and practice.

Adolescent girls engaged in sports reported significantly higher scores on Fit Overvaluation, Fit Drive, and the total FIIT compared with non-sport-involved peers, with the largest difference emerging for Fit Drive (Cohen’s d = −0.55). Sport participation may encourage girls to value their bodies for their capabilities and physical competence [42] while simultaneously increasing the personal importance attached to being lean, toned, and physically fit. In many contemporary sport contexts, especially aesthetic, performance-related and appearance-related body ideals are closely intertwined, with fitness, discipline, and athletic competence often serving as markers of both sporting success and physical attractiveness [62,63]. Consequently, sport-involved girls may place greater value on attaining a fit body and engage more frequently in behaviours aimed at achieving this ideal, resulting in elevated Fit Overvaluation and Fit Drive scores. At the same time, the absence of group differences in Fit Idealisation suggests that the desire for a fit body is widespread among adolescent girls regardless of sport participation status, likely reflecting the pervasive influence of stereotyped body ideal messages disseminated through social cultural contexts [21,64,65].

All FIIT subscales were significantly and positively associated with muscular internalization, body dissatisfaction, and disordered eating symptoms, consistent with the hypothesised associations with related constructs and outcomes. The strongest zero-order correlations in the present study were observed between Fit Idealisation and disordered eating (r = 0.60) and between Fit Idealisation and body dissatisfaction (r = 0.50). These findings are broadly consistent with those reported by Uhlmann et al. [36], who found moderate-to-strong positive associations between FIIT scores and theoretically related constructs, including thin and muscular ideal internalization, objectified body consciousness, drive for thinness, appearance-oriented exercise motives, and social comparison. Consistent with the present findings, previous studies have also linked fit ideal internalization to more negative body image [33,66,67].

Negative associations were observed between all FIIT subscales and body appreciation as measured by the BAS-2 (r = −0.19 to −0.37) consistent with the theoretically expected relationship between fit ideal internalisation and positive body image. These findings suggest that girls who strongly internalise the fit ideal are less likely to appreciate and respect their bodies unconditionally. This interpretation is consistent with the theory proposing that appearance ideal internalization undermines positive body image by orienting self-evaluation toward adherence to external appearance standards rather than appreciation and acceptance of the body as it is [68]. According to this perspective, health-promoting behaviours such as physical activity and healthy eating are viewed as expressions of body care and respect rather than as means of achieving a socially prescribed appearance. Consequently, girls who strongly internalise the fit ideal may become more likely to engage in these behaviours for appearance-related reasons, which may be incompatible with the unconditional body acceptance that characterises positive body image. Previous research similarly demonstrated that thin ideal internalization is weakly but significantly negatively associated with body appreciation among young women [69].

Associations between the FIIT subscales and autonomous exercise motivation were generally weak, providing evidence that fit ideal internalization is conceptually distinct from self-determined motivation for physical activity. In particular, no significant association was observed between Fit Drive and autonomous exercise motivation. This finding suggests that behavioural efforts aimed at achieving a lean and toned body are not necessarily driven by enjoyment, personal interest, or personally endorsed health values. Rather, they may be motivated by appearance-related concerns and external standards of attractiveness. This interpretation is consistent with the original validation study, in which Fit Drive was strongly associated with appearance motives for exercise but only weakly associated with health motives [36].

Further, a weak negative association was observed between Fit Overvaluation and autonomous exercise motivation (r = −0.18) consistent with the theoretically expected pattern. This finding is theoretically meaningful, as the Fit Overvaluation subscale reflects beliefs that possessing a fit body is associated with social approval and respect from others. From a Self-Determination Theory perspective [70], such beliefs are conceptually close to controlled forms of motivation, particularly introjected regulation, in which behaviour is driven by the desire for approval from others. Consequently, stronger fit overvaluation may be associated with lower levels of autonomous exercise motivation. Similarly, the association between Fit Idealization and autonomous exercise motivation was very weak (r = −0.09). This may be because Fit Idealization primarily reflects admiration of and desire for a lean and toned physique rather than the motivational reasons for engaging in physical activity. Although the subscale includes one item referring to guilt when not engaging in behaviours that facilitate attainment of the fit ideal, most items assess cognitive and affective investment in the ideal itself rather than exercise motivation. Overall, the pattern of associations provides further support for the construct validity of the FIIT. Whereas Fit Drive appeared to be independent of autonomous exercise motivation, Fit Idealization and particularly Fit Overvaluation showed weak inverse associations, suggesting that stronger endorsement of the fit ideal may be accompanied by lower levels of self-determined motivation for physical activity.

FIIT scores showed weak positive correlations with BMI (r = 0.10–0.18), indicating that girls with higher BMI tended to report slightly greater fit ideal internalisation. Although the magnitude of these associations was weak, the findings are consistent with the notion that girls whose bodies deviate more from the culturally promoted lean and toned ideal may be somewhat more likely to endorse and internalize this ideal. At the same time, the small effect sizes indicate that fit ideal internalization is evident across the BMI spectrum and is not strongly determined by body size. These findings are also broadly consistent with previous research showing that higher BMI among women is associated with greater thin ideal internalization, whereas no significant association has been observed with muscular ideal internalization [40]. Given that the fit ideal incorporates elements of both thinness and toned muscularity, the weak positive associations observed in the present study may primarily reflect the thinness-related component of the construct.

The hierarchical regression analyses demonstrated that the FIIT subscales explained significant additional variance in disordered eating (ΔR^2^ = 0.092, *p* < 0.001) beyond BMI, body dissatisfaction, and muscular internalization, which together accounted for 39.3% of the variance in disordered eating in Step 1. These findings indicate that fit ideal internalization contributes unique explanatory value beyond established correlates of disordered eating, supporting the incremental validity of the FIIT. The results further suggest that fit ideal internalization captures aspects of appearance-related cognition and behaviour that are not fully accounted for by body dissatisfaction, muscular internalization, or body mass alone, highlighting its relevance as a distinct correlate of disordered eating among adolescent girls. These findings are consistent with those of Uhlmann et al. [36], who reported that FIIT scores explained unique variance in eating-related pathology beyond existing measures of appearance ideal internalization [36].

Among the FIIT subscales, only FIIT Idealization emerged as a unique and significant correlate of disordered eating in the final model, whereas Fit Overvaluation and Fit Drive did not demonstrate significant independent contributions when all three subscales were entered simultaneously. This finding suggests that the cognitive–affective desire for congruence with the fit ideal may be particularly relevant to disordered eating symptoms among adolescent girls. The absence of unique effects for Fit Overvaluation and Fit Drive most likely reflects the substantial overlap among the FIIT dimensions, as evidenced by their strong intercorrelations (r = 0.67–0.75 in the present study; r = 0.59–0.66 in Uhlmann et al. [36], rather than a lack of theoretical or empirical relevance. Indeed, both subscales were significantly associated with disordered eating at the bivariate level, indicating that they remain important components of fit ideal internalization. Taken together, these findings support the conceptualisation of fit ideal internalization as a multidimensional construct comprising related yet distinguishable dimensions, while also highlighting the central role of FIIT Idealization in explaining variance in disordered eating in adolescent girls.

Mean FIIT scores in the present sample were moderate across all subscales (range: 2.39–2.68 on a 1–5 scale), with FIIT Idealisation yielding the highest mean, followed by Fit Drive and Fit Overvaluation. For comparison, Uhlmann et al. [36] reported mean item-level scores of approximately 3.29 for FIIT Idealisation, 2.93 for Fit Overvaluation, and 2.96 for Fit Drive in their Australian university student samples. Although mean scores in the present study were somewhat lower, particularly for Fit Overvaluation, direct comparisons should be interpreted cautiously given differences in age, cultural context, and sample characteristics between the studies. Notably, FIIT Idealisation exhibited the highest mean score in both studies, suggesting that the desire to attain the fit ideal may represent the most salient component of fit ideal internalization across diverse female populations.

Some strengths of the present study include the use of a two-step analytic strategy to determine the FIIT factor structure and the examination of measurement invariance across sport participation groups. Several limitations should nevertheless be acknowledged. First, the sample was restricted to adolescent girls from Lithuanian secondary schools. Although this demographic focus was theoretically motivated by the elevated vulnerability of adolescent girls to fit ideal exposure and eating pathology onset [37], it limits the generalizability of the findings to other populations. Uhlmann et al. [36] explicitly called for examination of the FIIT in younger and non-Australian samples, and the present study extends this work. Future research should examine the FIIT in boys, gender-diverse adolescents, adult women across different age groups, and clinical populations to further establish the breadth of its applicability. Second, although measurement invariance was established across sport participation groups, invariance across other potentially relevant subgroups, such as BMI category and socioeconomic status, was not examined and warrants attention in future research. Another limitation of the present study is that compulsive exercise was not assessed because no validated Lithuanian-language instrument was available. The absence of this important variable prevented us from distinguishing between healthy and compulsive forms of exercise engagement. Including this construct would have provided a more comprehensive understanding of the behavioural correlates of fit ideal internalization and would have enabled examination of the incremental validity of the FIIT in predicting compulsive exercise beyond established thin and muscular appearance internalization constructs. Third, the cross-sectional design did not permit a second administration of the FIIT and therefore precluded examination of test–retest reliability. Future studies would benefit from investigating the temporal stability of FIIT scores and from incorporating additional theoretically relevant constructs, such as body functionality appreciation and mindfulness during physical activity, to further examine the relationships between FIIT and other theoretically relevant constructs.

## 5. Conclusions

The present study provides evidence that the Lithuanian version of the FIIT is a valid and reliable instrument for assessing fit ideal internalization among adolescent girls. By extending previous validation research to a younger, non-Australian population, the present study contributes to the growing body of literature highlighting the role of fit ideal internalization in contemporary body image among adolescent girls. The findings further suggest that fit ideal internalization represents a meaningful and multidimensional construct that is associated with body image concerns and disordered eating beyond traditional indicators such as body dissatisfaction and muscular ideal internalization. As such, the FIIT may serve as a valuable tool for future research examining the role of contemporary appearance ideals in adolescent girls’ health and well-being.

## Figures and Tables

**Table 1:** Descriptive statistics of the Fit Ideal Internalisation Test (FIIT) subscales and total score (*n* = 1067).

Scales	Mean	SD	Median	Skewness	Kurtosis	Range
Fit Idealization	2.68	1.10	3.00	−0.02	−0.86	1–5
Fit Overvaluation	2.39	1.05	2.50	0.25	−0.69	1–5
Fit Drive	2.39	1.06	2.50	0.16	−0.83	1–5
FIIT Total	2.51	0.97	2.70	0.03	−0.65	1–5

Note. FIIT—Fit Ideal Internalisation Test; SD—standard deviation.

**Table 2:** Exploratory factor analysis of the Fit Ideal Internalisation Test (FIIT) in the first split-half subsample (*n* = 534).

Items	Fit Overvaluation	Fit Idealization	Fit Drive	h^2^
FIIT 1		0.463		0.570
FIIT 2		0.844		0.714
FIIT 3		0.917		0.823
FIIT 4		0.946		0.827
FIIT 5		0.874		0.781
FIIT 6		0.839		0.824
FIIT 7		0.611		0.749
FIIT 8		0.411		0.552
FIIT 9	0.769			0.751
FIIT 10	0.900			0.770
FIIT 11	0.726			0.737
FIIT 12	0.908			0.786
FIIT 13	0.804			0.737
FIIT 14	0.753			0.743
FIIT 15	0.915			0.821
FIIT 16	0.637			0.680
FIIT 17			0.746	0.658
FIIT 18			0.953	0.865
FIIT 19			0.875	0.798
FIIT 20			0.742	0.702

Note. h^2^—communalities.

**Table 3:** Fit indices for confirmatory factor analysis and measurement invariance models of the Fit Ideal Internalisation Test (FIIT) across sport participation groups in the second split-half subsample (*n* = 533).

Model	SBχ^2^	*df*	Robust CFI	Robust TLI	Robust RMSEA	SRMR	∆CFI	∆RMSEA	∆SRMR
Initial CFA model	511.4	167	0.939	0.931	0.086	0.052	–	–	–
Final CFA model	435.6	163	0.952	0.945	0.077	0.047	–	–	–
Configural	638.2	326	0.944	0.935	0.084	0.050	–	–	–
Metric	719.7	343	0.942	0.936	0.083	0.053	0.002	0.001	0.003
Scalar	759.4	360	0.940	0.937	0.083	0.053	0.002	0.000	0.000
Strict	832.0	380	0.930	0.930	0.087	0.053	0.010	0.004	0.000

Note. SB = Satorra-Bentler; *df* = degrees of freedom; CFI = comparative fit index; RMSEA = Steiger-Lind root mean square error of approximation; SRMR = standardised root mean square residual.

**Table 4:** Differences in Fit Ideal Internalisation Test subscales, and total scores between girls engaged and not engaged in sports, m ± SD (*n* = 1067).

Scale	Engagement in Sports		*p*	Cohen’s d
	No (*n* = 266)	Yes (*n* = 801)		
Fit Idealization	2.59 ± 1.20	2.71 ± 1.06	0.140	−0.11
Fit Overvaluation	2.27 ± 1.11	2.43 ± 1.03	0.035	−0.16
Fit Drive	1.96 ± 1.06	2.53 ± 1.02	<0.001	−0.55
FIIT Total	2.34 ± 1.02	2.56 ± 0.95	0.001	−0.24

Note. FIIT—Fit Ideal Internalisation Test; m—mean, SD—standard deviation.

**Table 5:** Correlations between FIIT subscales and study variables (*n* = 1063).

Variables	Fit Idealization	Fit Overvaluation	Fit Drive
Body mass index	0.18 **	0.16 **	0.10 **
SATAQ-4: Muscular Internalisation	0.45 **	0.38 **	0.47 **
EDI-3: Body Dissatisfaction	0.50 **	0.37 **	0.32 **
EDEQ-6	0.60 **	0.45 **	0.38 **
BAS-2	−0.37 **	−0.27 **	−0.19 **
Relative Autonomy Index	−0.09 **	−0.18 **	−0.003

Note. SATAQ-4—Social Attitudes Towards Appearance Questionnaire 4, EDI-3—Eating Disorder Inventory 3, EDE-Q 6—Eating Disorder Examination Questionnaire 6, BAS-2—Body Appreciation Scale 2, ** *p* < 0.01.

**Table 6:** Hierarchical regression analysis examining the incremental validity of Fit Ideal Internalisation Test subscales in relation to disordered eating symptoms in adolescent girls (*n* = 1063).

Measures	B	SE B	β	*p*
Step 1				
Body mass index	0.036	0.011	0.081	0.002
EDI-3: Body Dissatisfaction	0.749	0.036	0.546	<0.001
SATAQ-4: Internalisation muscular	0.241	0.034	0.172	< 0.001
Model summary: R^2^ = 0.393, adj. R^2^ = 0.391, F = 228.70, *p* < 0.001				
Step 2				
Body mass index	0.027	0.011	0.061	0.010
EDI-3: Body Dissatisfaction	0.525	0.037	0.382	<0.001
SATAQ-4: Muscular Internalisation	0.045	0.036	0.032	0.217
Fit Idealization	0.532	0.051	0.401	<0.001
Fit Overvaluation	0.034	0.052	0.025	0.511
Fit Drive	−0.074	0.047	−0.054	0.119
Model summary: R^2^ = 0.486, adj. R^2^ = 0.483, ∆R^2^ = 0.092, ∆F = 63.23, *p* < 0.001				

Note. B—unstandardized, β—standardised regression coefficient, SE—standard error, SATAQ-4—Social Attitudes Towards Appearance Questionnaire 4, EDI-3—Eating Disorder Inventory 3.

## Data Availability

The dataset used in this study is provided in the Supplementary Materials.

## Supplementary Materials

The following supporting information can be downloaded at: https://www.mdpi.com/article/10.3390/nu18193248/s1, Table S1: Dataset containing the study variables used in the present analyses.

## Appendix A. Lithuanian Version of the Fit Ideal Internalization Test (FIIT)/Sportiško Kūno Idealizavimo Klausimynas

Atsakymų variantai: 1—visiškai nesutinku, 2—labiau nesutinku, 3—nei sutinku, nei nesutinku, 4—labiau sutinku, 5—visiškai sutinku.

1. Dažnai jaučiuosi susirūpinęs/usi pažanga, kurią darau siekdamas/-a tobulai liekno ir tvirto kūno (tvirtas kūnas—su išryškėjusiais stipriais raumenimis)
2. Nuolat lyginu savo kūną su kitų žmonių, kurių kūnai liekni ir tvirti
3. Visą laiką mane persekioja įkyrios mintys apie mano kūno dalis, kurios nėra labai lieknos ir tvirtos
4. Visą laiką svajoju apie tai, kaip atrodyčiau turėdamas/a labai liekną ir tvirtą kūną
5. Jaučiu kaltę, kai nedarau dalykų, kurie padeda man siekti liekno ir tvirto kūno (pvz. nesilaikau dietos, nesimankštinu)
6. Daug galvoju apie tai, ką galėčiau padaryti, kad mano kūnas atrodytų lieknas ir tvirtas
7. Esu apsėstas/a idėjos turėti kūną, kuris yra lieknas ir tvirtas
8. Kad pasiekčiau kūną, kokio noriu, svarbu derinti griežtą mitybos ir mankštinimosi režimą
9. Labai lieknas ir tvirtas kūnas yra geras būdas parodyti žmonėms, kad tu kontroliuoji savo gyvenimą
10. Liekno ir tvirto kūno turėjimas yra geras būdas užsitarnauti kitų žmonių pagarbą
11. Kuo daugiau darau dalykų (pvz., mankštinuosi, laikausi dietos), kad mano kūnas atrodytų lieknas ir tvirtas, tuo labiau kiti žmonės mane vertina
12. Lieknas ir tvirtas kūno sudėjimas pasako kai ką svarbaus apie tai, kas tu esi kaip žmogus
13. Liekno ir tvirto kūno palaikymas yra geras būdas parodyti žmonėms, koks esu darbštus/-i
14. Liekno ir tvirto kūno turėjimas leidžia jaustis sėkmingu gyvenime
15. Jei galiu turėti liekną ir tvirtą kūną, tai sako kai ką gero apie mane kaip apie žmogų
16. Jei turėčiau itin liekną ir labai tvirtą kūną, būčiau populiaresnis/ė tarp savo bendraamžių
17. Leidžiu laiką darydamas/a dalykus (pvz., mankštinuosi, laikausi dietos, vartoju maisto papildus), kad išvystyčiau matomus raumenis
18. Leidžiu laiką darydamas/a dalykus (pvz., mankštinuosi, laikausi dietos, vartoju maisto papildus), kad užsitikrinčiau, jog mano kūnas atrodo lieknas ir tvirtas
19. Leidžiu laiką darydamas/a dalykus (pvz., mankštinuosi, laikausi dietos, vartoju maisto papildus), kad užsitikrinčiau, jog mano kūnas atrodo labai lieknas
20. Leidžiu laiką darydamas/a dalykus (pvz., mankštinuosi, laikausi dietos, vartoju maisto papildus), kad sudeginčiau riebalus
